# Estimating the size of the solution space of metabolic networks

**DOI:** 10.1186/1471-2105-9-240

**Published:** 2008-05-19

**Authors:** Alfredo Braunstein, Roberto Mulet, Andrea Pagnani

**Affiliations:** 1 Politecnico di Torino, Corso Duca degli Abruzzi 34, I-10129, Torino, Italy; 2 ISI Foundation Viale Settimio Severo 65, Villa Gualino, I-10133 Torino, Italy; 3 "Henri-Poincaré-Group" of Complex Systems and Department of Theoretical Physics, Physics Faculty, University of Havana, La Habana, CP 10400, Cuba

## Abstract

**Background:**

Cellular metabolism is one of the most investigated system of biological interactions. While the topological nature of individual reactions and pathways in the network is quite well understood there is still a lack of comprehension regarding the global functional behavior of the system. In the last few years flux-balance analysis (FBA) has been the most successful and widely used technique for studying metabolism at system level. This method strongly relies on the hypothesis that the organism maximizes an objective function. However only under very specific biological conditions (*e.g*. maximization of biomass for *E. coli *in reach nutrient medium) the cell seems to obey such optimization law. A more refined analysis not assuming extremization remains an elusive task for large metabolic systems due to algorithmic limitations.

**Results:**

In this work we propose a novel algorithmic strategy that provides an efficient characterization of the whole set of stable fluxes compatible with the metabolic constraints. Using a technique derived from the fields of statistical physics and information theory we designed a message-passing algorithm to estimate the size of the affine space containing all possible steady-state flux distributions of metabolic networks. The algorithm, based on the well known Bethe approximation, can be used to approximately compute the volume of a non full-dimensional convex polytope in high dimensions. We first compare the accuracy of the predictions with an exact algorithm on small random metabolic networks. We also verify that the predictions of the algorithm match closely those of Monte Carlo based methods in the case of the Red Blood Cell metabolic network. Then we test the effect of gene knock-outs on the size of the solution space in the case of *E. coli *central metabolism. Finally we analyze the statistical properties of the average fluxes of the reactions in the *E. coli *metabolic network.

**Conclusion:**

We propose a novel efficient distributed algorithmic strategy to estimate the size and shape of the affine space of a non full-dimensional convex polytope in high dimensions. The method is shown to obtain, quantitatively and qualitatively compatible results with the ones of standard algorithms (where this comparison is possible) being still efficient on the analysis of large biological systems, where exact deterministic methods experience an explosion in algorithmic time. The algorithm we propose can be considered as an alternative to Monte Carlo sampling methods.

## Background

Cellular metabolism is a complex biological problem. It can be viewed as a chemical engine that transforms available raw materials into energy or into the building blocks needed for the biological function of the cells. In more specific terms a metabolic network is indeed a processing system transforming *input *metabolites (nutrients), into output metabolites (amino acids, lipids, sugars etc.) according to very strict molecular proportions, often referred as stoichiometric coefficients of the reactions.

Although the general topological properties of these networks are well characterized [1-3], and non-trivial pathways are well known for many species [4] the cooperative role of these pathways is hard to comprehend. In fact, the large sizes of these networks, usually containing hundreds of metabolites and even more reactions, makes the comprehension of the principles that govern their global function a challenging task. Therefore, a necessary step to achieve this goal is the use of mathematical models and the development of novel statistical techniques to characterize and simulate these networks.

It is well known that under evolutionary pressure, prokaryote cells like *E. coli *behave optimizing their growth performance [5]. Flux Balance Analysis (FBA) provides a powerful tool to predict the optimal growth and production fluxes, but is not reliable about phenotypic state the cell will acquire. This is mainly due to the fact that among the infinitely many potential network states compatible with the stoichiometric constraints, FBA chooses a single one whose biological meaning is at least questionable under generic external conditions. FBA maximizes a linear function (usually the growth rate of the cell) subject to biochemical and thermodynamic constraints [6]. On the other hand, cells with genetically engineered knockouts or bacterial strains that were not exposed to evolution pressures, need not to optimize their growth. In fact, the method of minimization metabolic adjustment [7] has shown that knockout metabolic fluxes undergo a minimal redistribution with respect to the flux configuration of the wild type. Yet, in more general situations, the results are unpredictable, therefore, a tool to characterize the shape and volume of the whole space of possible phenotypic solutions must be welcome.

So far, apart from exact algorithms evaluating the volume of the space of possible solutions, that are unsuitable for analyzing metabolic networks larger than some dozens metabolites [8,9], the best technique allowing for such a characterization is based on Monte Carlo sampling (MCS) of the steady-state flux space [10-14]. This method is known to perform very well on intermediate size metabolic networks (up to a hundred of metabolites) [10,11] where different strategies of MCS have been implemented giving comparable results. Some improved variant of MCS seems to perform well also on organism-wide where the number of variables is in the range order of a thousand [13,14]. Although MCS has in general some intrinsically associated problems, mainly due to the fact that the convergence (or mixing) time is hard to assess and often is exponential, in the case of polytope volume estimations it turns out that sampling strategies such as *hit and run *have mixing times that scale only polynomially with system size [15]. However in many concrete cases a practical problem is to give a precise condition for convergence therefore we believe that an alternative independent technique could be more than welcome even in the cases where MCS is applicable.

As a concrete step toward an efficient characterization of the set of fluxes compatible with the stoichiometric constraints, we propose a novel message-passing technique derived from the field of statistical physics and information theory.

### Mathematical Model

As already mentioned, a metabolic network is an engine that converts metabolites into other metabolites through a series of intra-cellular intermediate steps. The fundamental equation characterizing all functional states of a reconstructed biochemical reaction network is a mass conservation law that imposes simple linear constraints between the incoming and outcoming fluxes at any chemical reaction:

(1) $$\frac{\partial \rho }{\partial t}=i+\overset{‸}{S}\cdot \nu -o$$

where *ρ *is the vector of the *M *metabolite concentrations in the network. **i **(**o**) is the input (output) vector of fluxes, and ***ν ***are the reaction fluxes governed by the *M *× *N *stoichiometric linear operator $\overset{‸}{S}$ (usually named stoichiometric matrix) encoding the coefficient of the *M *intra-cellular relations among the *N *fluxes.

As long as just steady-state cellular properties are concerned one can assume that a variation in the concentration of metabolites in a cell can be ignored and considered as constant. Therefore in case of fixed external conditions one can assume metabolites (quasi) stationarity and consequently the *lhs *of 1 can be set to zero. Under these hypotheses the problem of finding the metabolic fluxes compatible with flux-balance is mathematically described by the linear system of equations

(2) $$\overset{‸}{S}\cdot \nu =o-i\equiv b$$

where **b **is the net metabolite uptake by the cell. Without loss of generality we can assume that the stoichiometric matrix $\overset{‸}{S}$ has full rows rank, *i.e*. that rank($\overset{‸}{S}$) = *M*, since linearly dependent equations can be easily identified and removed. Given that the number of metabolites *M *is lower than the number of fluxes *N *the subspace of solutions is a (*N-M*)-dimensional manifold embedded in the *N*-dimensional space of fluxes. In addition, the positivity of fluxes, together with the experimentally accessible values for the maximal fluxes, limit further the space of feasible solutions. This fact may be expressed by the following inequalities:

(3) **0 **≤ ***ν ***≤ ***ν***^max^

in such a way that together, 2 and 3, define the convex set of all the allowed time-independent phenotipic states of a given metabolic network.

### Sub-dimensional volumes

Mathematically speaking, the space of feasible solutions consistent with the Equations 2 is an affine space *V *⊂ ℝ^*N *^of dimension *N *- *M*. The set of inequalities 3 then defines a convex polytope Π ⊂ *V *that, from the metabolic point of view, may be considered as the allowed configuration space for the cell states. The main goal of this work is computation of the volume of this space of solutions and of certain subspaces of it. Although conceptually simple, the notion of sub-dimensional volume like that of Π requires some new definitions.

**Figure 1:**
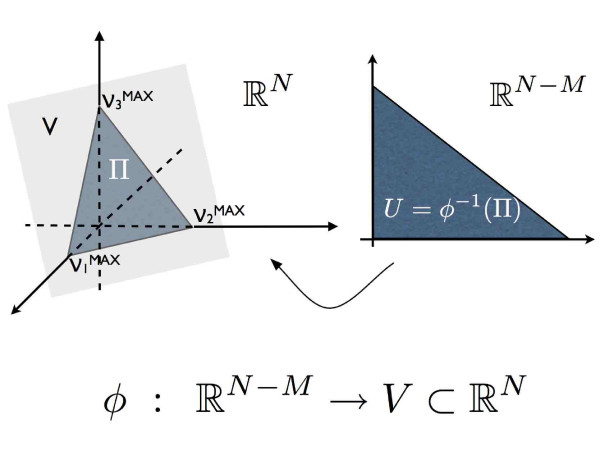
**Embedding**. Sketch of the the parameterization *ϕ *= *ℝ*^*N - M *^→ *V *⊂ *ℝ*^*N*^, in a simple case where *N *= 3 and *M *= 1. The mass balance equations defined in Equation 2 define the hyperplane *V *and the set of inequalities defined in Equation 3 restrict *V *to polytope Π indicated as the grey triangle in the left part of the figure. The application *ϕ *map the *full-dimensional *counter-image of Π (the grey triangle on the right indicate with *U*) onto Π.

Consider any linear parameterization *ϕ *: ℝ^*N*-*M *^→ *V *⊂ ℝ^*N *^(see explicative scheme in Figure 1). A popular choice for *ϕ *is, for instance, the inverse of the so called *lexicographical *projection *i.e*., the projection over the first *N-M *coordinates such that its restriction to *V *has an inverse. Being *ϕ *linear, the (*N - M*) × *N *Jacobian matrix $\overset{‸}{\lambda }$ is constant and coincides with the matrix of *ϕ *in the canonical bases. Denoting *λ *= det ${\left({\overset{‸}{\lambda }}^{\text{†}}\overset{‸}{\lambda }\right)}^{\frac{1}{2}}$, the Euclidean metric in ℝ^*N *^induces a measure on *V *(which does not depend on *ϕ*):

(4) $$\int_{V}f(\nu )d\nu \equiv \lambda \int f(\varphi (u))du$$

allowing to compute the volume of our polytope

(5) $$vol_{V}(\Pi )\equiv \int_{V}1_{\Pi }(\nu )d\nu =\lambda \int 1_{\varphi^{-1}(\Pi )}(u)du$$

where **1**_Π _(·) is the indicator function of the set Π. It is worth pointing out that given the linear structure of the metabolic equations, the determinant of the mapping is a (scalar) constant. Although the coefficient *λ *could be explicitly calculated, it turns out that as far as only relative volume quantities are concerned, as in the case of the *in silico *flux knock-outs introduced below, this term factors out and therefore we will drop it from the rest of the computation.

### Probabilistic framework

The problem of describing the polytope Π can be cast into a probabilistic framework. We define the probability density P as:

(6) $$P(\nu )=vol_{V}{\left(\Pi \right)}^{-1}1_{\Pi }(\nu )$$

**Figure 2:**
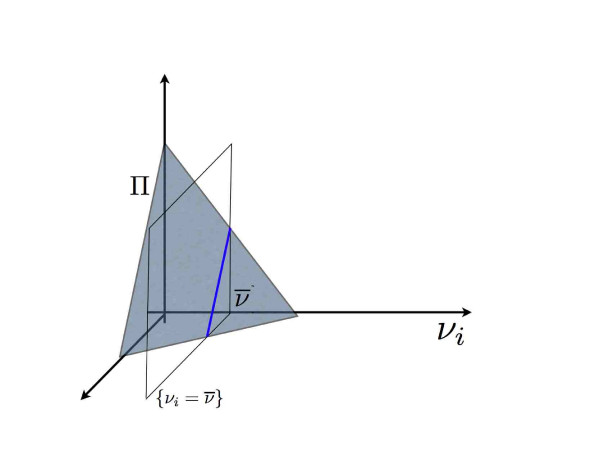
**Marginal probability**. Geometrical interpretation of the marginal probability distributions: the marginal probability at point *ν*_*i *_= $\bar{\nu }$ is proportional to length of the blue segment which is the intersection between the polytope Π (the grey triangle) and the plane *ν*_*i *_= $\bar{\nu }$.

Marginal flux probabilities over a given set of fluxes are obtained by integrating out all remaining degrees of freedom. In particular we can define single flux marginal probability densities as integrals on the affine subspace *W *= *V *∩ {*ν*_*i *_= $\bar{\nu }$}

(7) $$P_{i}(\nu_{i})=\int_{W}P(\nu )\prod_{j\neq i}d\nu_{j}=\frac{vol_{W}(\Pi \cap W)}{vol_{V}(\Pi )}$$

where the normalization term **vol**_*W *_(Π ∩ *W*) is the (sub dimensional) volume of the intersection between the polytope Π and the hyperplane {*ν*_*i *_= $\bar{\nu }$} as displayed schematically in Figure 2 where the marginal probability at point *ν*_*i *_= $\bar{\nu }$ is proportional to length of the blue segment which is the intersection between the polytope Π and the plane *ν*_*i *_= $\bar{\nu }$

### Approximate volume computation

¿From a computational point of view, the problem of the exact computation of the volume of a polytope with current methods requires the enumeration of all its vertexes. The vertex enumeration problem is #*P*-hard [16,17], but even the problem of computing the volume, given the set of all vertexes is a big computational challenge. Various algorithms exist for calculating the exact volume of a polytope from its vertexes (for a review see [18]), and many software packages are available in the Internet. Computational limitations restrict however exact algorithmic strategies to cope with polytopes in relatively few dimensions (*e.g. N - M *around 10 or so). To overcome such severe limitations we will introduce a very efficient approximate computational strategy that will allow us to compute the volume and the shape of the space of solutions for real-world metabolic networks.

We will adopt the following three steps strategy:

• We discretize the problem *a la Riemann *considering an a *N*-dimensional square lattice whose elementary cell is of size *ε*^*N*^. The approximated volume is then proportional to the number of cells intersecting the polytope Π. Of course the smaller *ε *the better is the approximation.

• We consider an integer constraint satisfaction problem where each of the mass-balance equations set a hard constraint over the involved discretized fluxes.

• We solve the constraint satisfaction problem using a message-passing algorithm called Belief Propagation.

#### Discretization

**Figure 3:**
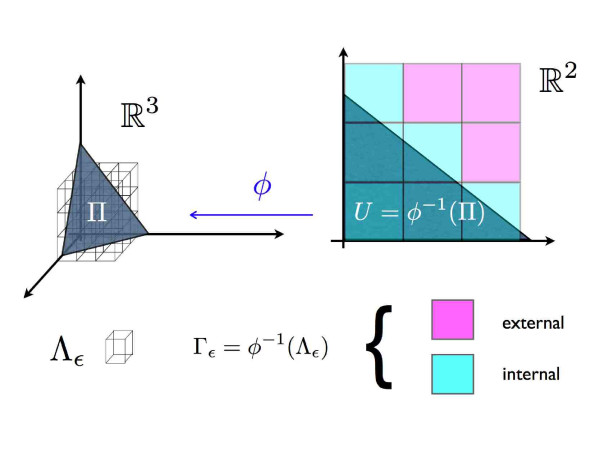
**Tiling**. Discretization of the volume: in the left part of the figure we display the the regular orthogonal grid Λ_*ε *_of side *ε *partitioning ℝ^*N*^. The counter-image of Λ_*ε *_via *ϕ *is given by the the grid Γ_*ε *_. Let number of Γ_*ε *_squares intersecting *ϕ*^-1^(Π) is proportional to the number of Λ_*ε *_cubes intersecting Π. The smaller the *ε *the better the intersection of the grid Λ_*ε *_with the polytope Π will approximate Π.

Consider the regular orthogonal grid Λ_*ε *_of side *ε *partitioning ℝ^*N *^as in the simple sketch of Figure 3. This grid maps via *ϕ*^-1 ^into a partition Γ_*ε *_of *ϕ*^-1^(Π). The number of cells $N_{\varepsilon }$_*ε *_of Λ_*ε *_intersecting Π is proportional to the numbers of cells of Γ_*ε *_intersecting *ϕ*^-1^(Π). Finally, the volume in Eq. 5 is proportional to lim_*ε *→ 0_*ε*^*N*-*M*^$N_{\varepsilon }$_*ε *_. For any given *ε *one we have then defined discrete variables *ν*_*i *_∈ {0,1,..., $q_{i}^{\max}$}, for $q_{i}^{\max}$ equal to the integer part of $q^{\max}\times \nu_{i}^{\max}$, where the integer *q*^max ^is the granularity of the approximation.

#### The constraint satisfaction problem

When dealing with integer coefficients *s*_*i, a*_, as the ones appearing in *normal *stoichiometric relations, the discrete version of Eqs. 2 close in the set of positive integers defining a constraint satisfaction problem. The *ε*-approximation of the volume is then the number of elementary cells that are solution to the discretized mass-balance equations. It is interesting to note that in the case of fractional stoichiometric relations one can multiply all terms for the minimum common multiple of all denominators, getting an equivalent mass-balance equation with integer coefficients.

#### Belief Propagation

The integer constraint satisfaction is solved using Belief Propagation (BP), a local iterative algorithm that allows for the computations of marginal probability distributions. BP is exact on trees, and perform reasonably well on locally tree-like structures [19-22]. This approximation scheme allows for the computation of the logarithm of the number of solutions via the entropy that can be expressed in terms of flux marginals. We will give a detailed derivation of the equations in section Methods.

## Results and Discussion

### Performance on low dimensional systems

In this section we will analyze the performance of our algorithm against an exact algorithm on low dimensional polytopes. Among the different packages available in the Internet, we have chosen LRS [8], a program based on the *reverse search *algorithm presented by Avis and Fukuda in [9] that can compute the volume of non-full dimensional polytopes. Actually, it computes the volume of the lexicographically smallest representation of the polytope, that for the benchmark used below, coincides with the conventional volume estimated by our algorithm.

We have devised a specific benchmark generating random diluted stoichiometric matrices at a given ratio *α *= *M/N *and fixed number of terms different from zero *K *in each of the reactions. All fluxes were constrained inside the hypercube 0 ≤ *ν*_*i *_≤ 1. As a general strategy we have calculated several random instances of the problem and measured the volume (entropy) of the polytope using the LRS and BP algorithm. In particular, we have first generated 1000 realizations of random stoichiometric matrices with *N *= 12, *M *= 4. Note that *N *= 12 is around the maximum that allows simulations with LRS in reasonable time (around one hour per instance). For each polytope then we have computed the two entropies *S*_*LRS *_and *S*_*BP *_with both algorithms, fixing the same maximum value for the discretization *q*^max ^= 1024 for all fluxes.

**Figure 4:**
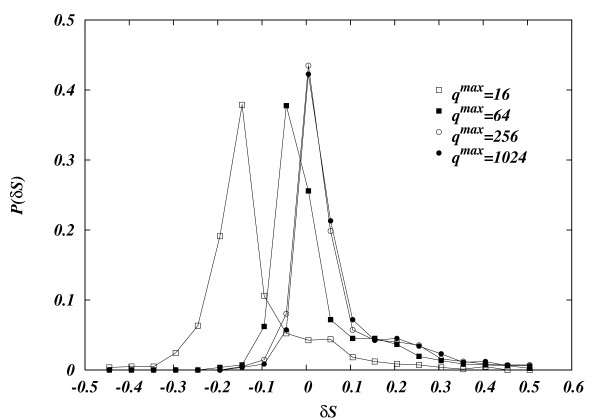
**Discrepance histogram**. Histograms of *δS *= (S_*BP *_- S_*LRS*_)/S_*LRS*_, where S_*LRS *_and S_*BP *_are the estimates of the logarithm of the volume computed using respectively the program LRS [8], and our message-passing algorithm. The measurement if *δS *are taken over a set of 1000 random realizations of a *N *= 12 and *M *= 4 stoichiometric matrix where each of the metabolites participates to *K *= 3 different reactions. The four histograms represent the distribution of the experimental frequencies of the measured *δS*. Measurements were taken at different values of of the discretization parameter *q*^max ^= 16, 64, 256, 1024. For larger values of *q *the distributions of the measured *δ*_*S *_peak around zero.

In Figure 4 we show how the quality of the BP measure is affected by the discretization, by displaying the histogram of the relative differences $\delta S=\frac{S_{BP}-S_{LRS}}{S_{LRS}}$ with an increasing number of bins per variable *q*^max ^= 16 , 64, 256, 1024. One can see how a finer binning of messages improves the quality of the approximation, seemingly converging to a single distribution of errors. It is expected that for larger *N *the histograms would peak around the true value: upon increasing the number of fluxes, loops become larger and the overall topology of the graph becomes more locally tree-like, validating the hypothesis behind the Bethe approximation. Unfortunately, the huge increase of computer time experimented in the calculation of the volumes using LRS made impossible to test systems large enough to make any reasonable scaling analysis.

**Figure 5:**
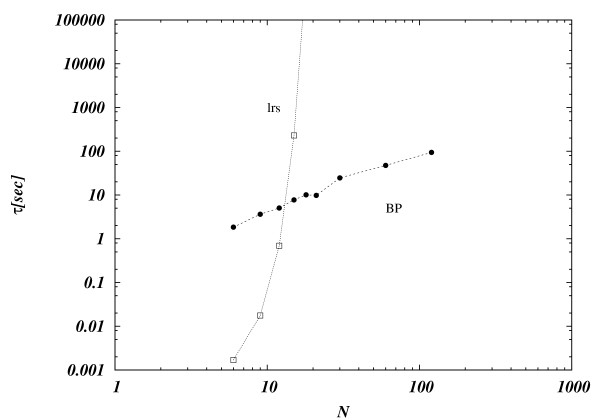
**Running time**. Logarithm of the running time *τ *expressed in seconds vs. *N *for LRS algorithm, and BP algorithm. Averages are taken over 5000 realizations of random stoichiometric matrices at each value of *N *except in the *N *= 12 case where we analyzed 500 realizations. LRS outperforms BP up to sizes *N *~12 where the running time of LRS explodes exponentially while BP maintains a modest almost-linear behavior.

Finally we address the issue of the computational complexity of the algorithm which is a crucial one if one is interested in approaching real world metabolic networks whose size typically is at least 50 times the size of the largest network that can be analyzed with exact algorithms. In Figure 5 we display the running time of both LRS and BP as a function of the number of fluxes N. Interesting, LRS outperforms BP up to sizes *N *~12 where the running time of LRS explodes exponentially while BP maintains a modest almost-linear behavior.

### Distribution of fluxes in Red Blood Cell

**Figure 6:**
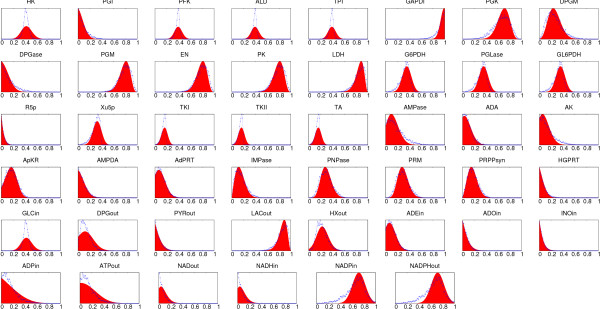
**Distribution of fluxes in Blood Cells**. Marginal probability distributions of the flux values for each of the 46 reaction in the red blood cell network computed using our message-passing algorithm (filled area) and the MC method (lines). Panels are arranged following the same sequence of figure 5 in [10]. The maximum values of the fluxes are all identical, $\nu_{i}^{\max}$ = 1.

**Figure 7:**
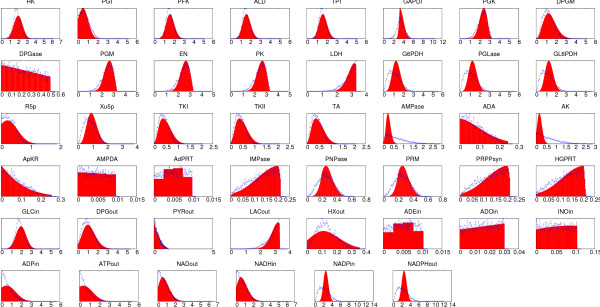
**Distribution of fluxes in Blood Cells**. Marginal probability distributions of the flux values for each of the 46 reaction in the red blood cell network computed using our message-passing algorithm (filled area) and the MC method (lines). Panels are arranged following the same sequence of figure 5 in [10]. The maximum values of the fluxes were taken from [10].

We used our BP algorithm and MCS [11] to obtain the marginal flux distributions for each of the reactions in the Red Blood Cell (RBC) metabolism taking the same stoichiometric matrix presented in [10,11] (see Additional file 1). The network contains 46 reactions and 34 metabolites. The first comparison of MCS with our BP algorithm is done setting all $\nu_{i}^{\max}$ to 1 (the resulting marginal probability distribution for the fluxes are displayed in Figure 6). The second comparison is done by setting all $\nu_{i}^{\max}$ to the values used in [10] (results are displayed in Figure 7). In both settings we compared BP with a set of 5000 feasible solutions generated by MCS, while for the BP algorithm we used a *q*^max ^= 2048. As it can be seen in the two cases, the predictions of both methods compare rather well. Assuming the accuracy of MCS, differences are probably due to small loops structures in the graph. We leave for a future work the issue of a more detailed comparison of both methods. However we really think that the emerging scenario of RBC metabolism captured by BP is analogous to that obtained by MCS both quantitatively and qualitatively.

The MCS method appears to be quite efficient (the authors of [11] reported in a similar network less then 30 seconds of computer computation in a Dell Dimension 8200 to obtain their distributions) while our algorithm converged to the same result in less than 3 seconds on a similar machine (Intel CPU 6600 2.40 *GHz*). Of course, no stringent statement can be done at this level about the comparative performance of the two algorithms. This positive result encourages us to face the problem of metabolism at organism-wide scale.

### Analysis of gene knock-out in *E. coli *central metabolism

In this section we study the influence of partial flux knock-out on the volume of the solution space. We concentrate on *E. coli central *metabolism [23]. The network has 62 metabolites, 104 reactions (75 internal reactions and 29 exchange fluxes). All reactions were considered irreversible following their nominal directions, and maximum flux rates were set to 1.

In this numerical experiment we first run BP on the unperturbed system measuring the volume of the space of solution *S*_0_. Then the maximum flux values are kept constant and equal to 1 for all the reactions but one (say reaction *ν*_*i*_). The partial knock-out effect on flux *i *is then obtained reducing repeatedly $\nu_{i}^{\max}$ and computing again the volume *S*_*KO*_($\nu_{i}^{\max}$) at each time until $\nu_{i}^{\max}$ = 0. In principle at each reduction step of $\nu_{i}^{\max}$ one should converge again the BP equations. In practice for most of the fluxes convergence is very fast since at each reduction of $\nu_{i}^{\max}$ the new stationary point will be in general close enough to the old one. However we have experimentally noted that the larger is the impact of a given flux on the volume of the space of solutions, the longer is the convergence time at each reduction step. Let us point out that doing so we are tacitly assuming that each knock-out is independent from the others although it is known that some reactions might be associated with the same enzyme. However there is no computational restriction to analyze multiple knock-outs. An analogous technique was presented in [11], where maximal fluxes were reduced to the value of 75% of their original maximal allowed flux to mimic enzymopathies.

**Figure 8:**
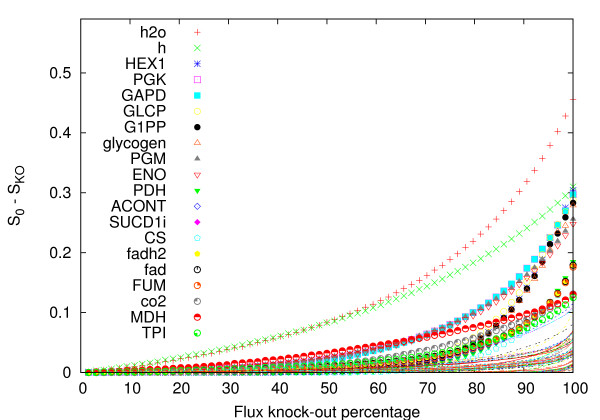
**Flux knock-out curves**. Flux knock-out impact on the volume of the space of solutions in *E. coli *central metabolism. On the x-axis we display the percentage of reduction of any given flux, and on the y-axis the relative volume difference with respect to the unperturbed system. We use upper-case keys for internal fluxes, and lower-case to exchange fluxes. We indicate keys only for the 20 fluxes having larger impact on the volume (dots) and we display the rest fluxes with thin scattered lines.

**Figure 9:**
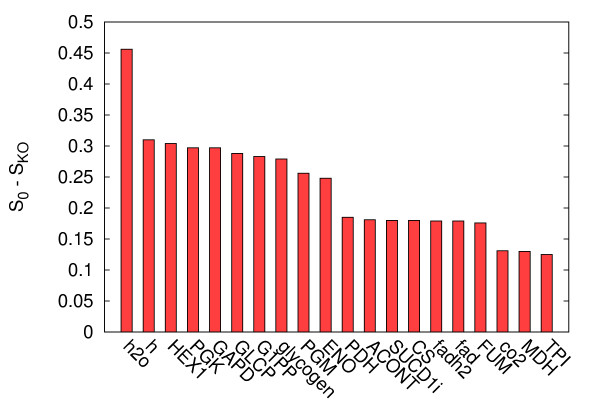
**Top impact flux knock-outs**. Impact of the 20 fluxes which have a larger impact on the volume of the space of solution. Here *S*_*KO *_= *S*_0 _- *S*_*KO *_($\nu_{i}^{\max}$ = 0)measures the difference of the volume of the unperturbed system and that modified by setting $\nu_{i}^{\max}$ = 0. In the x-axis we indicate the relative flux name using upper-case keys for internal fluxes, and lower-case to exchange fluxes.

In Figure 8 we display the whole set of *S*_0 _- *S*_KO_($\nu_{i}^{\max}$) vs. knock-out percentage curves. We can observe how heterogeneous is the impact of the different fluxes on the volume. Moreover one can observe how different curves may cross depending on the knock-out percentage displaying thus an intriguing scenario of differential flux-reduction impacts. Let us now concentrate on the 20 fluxes having larger impact on the space of solutions: in Figure 9 we display complete knock-outs values *S*_0 _*-S*_*KO*_($\nu_{i}^{\max}$ = 0). Focusing only on internal fluxes (the first two fluxes are indeed exchange fluxes of water and protons), one can observe that the first half of them basically compose the backbone of glycolysis showing little pathway redundancy in the network, while reactions like FUM, ACONT, SUC and SUCCD1i appear in the Krebs cycle and again show little pathway redundancy in the network (see Additional file 2).

**Figure 10:**
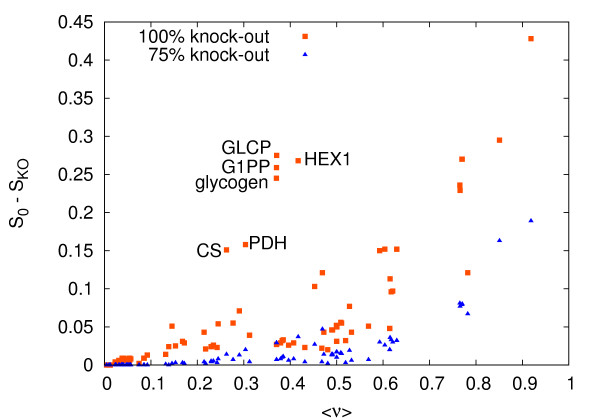
**Correlation of impact and value**. Change in entropy *S*_0 _- *S*_*KO *_after reaction knockouts and the average as a function of the average flux value ⟨*ν*⟩ of the unperturbed system. Red dots are relative to 75% knock-out and blue one to complete knock-out. The six red dots with average flux ~0.3 and entropy change larger than 0.15 are G1PP, GLCP, HEX1, CS, PDH, glycogen which are key check-points of central metabolism.

Finally in Figure 10 we display the correlations between the changes in the entropy for different reaction knock-outs and the average flux ⟨*ν*_*i*_⟩ = ∫P_*i *_(*ν*)*ν*d*ν *in the unperturbed network. At 75% knock-out, two kinds of regimes are divided by a clear threshold at *ν *~ 0.6: (i) for ⟨*ν*_*i*_⟩ < 0.6, S_0 _- S_*KO*_($\nu_{i}^{\max}$) has a small positive correlation with ⟨*ν*⟩, (ii) at larger average fluxes, correlations increase rapidly but with larger fluctuations. The presence of this threshold can be understood noting that reactions belonging to the linear (glycolysis) an circular (Krebs cycle) pathways are, in the wild cell, fast flux reactions, with average flux values larger than 0.5. An analogous scenario emerges in the case of a 100% knock-outs, but now fluctuation are wider, and also fluxes with intermediate average value start becoming important. This is the case for instance of the 6 large impact fluxes having average flux around 0.3. A closer inspection reveals that among them there is an exchange fluxes (glycogen) and 5 internal fluxes (G1PP, GLCP, HEX1, CS, PDH). The first three (G1PP, GLCP, HEX1) are the first steps of glycolysis, while PDH is the input of the Krebs cycle, and CS is a segment of the cycle strictly related to PDH. It is interesting that this peculiar behavior (large impact and relatively small average flux) are related to key check-points of central metabolism.

### *E. coli *metabolism at organism-wide scale

Finally we analyze the average fluxes distribution function in the metabolic network of *E. coli*. The network used contains, in its original format, 1035 reactions and 626 metabolites [23]. The network has been preprocessed using Gaussian elimination procedure: when a trivial mass-balance equation of the type *ν*_*i *_= *ν*_*j *_is present we eliminate variable *ν*_*i *_using elementary row operations. Doing so new trivial mass balance equations appear, an then we iterate the eliminations until all trivial mass-balance equations disappear. During Gaussian elimination, some mass balance equations become effective *dead-end *metabolite (*i.e *metabolites that are only consumed or produced). Fluxes participating to dead-end metabolites are set to zero at the beginning and removed from the system. The new set of equations is equivalent to the original one but with a lower number of fluxes. Reversible reactions have been considered using bidirectional signed fluxes. Using BP we are able to compute the marginal flux distributions in around 40 minutes, using *q*^max ^= 64.

**Figure 11:**
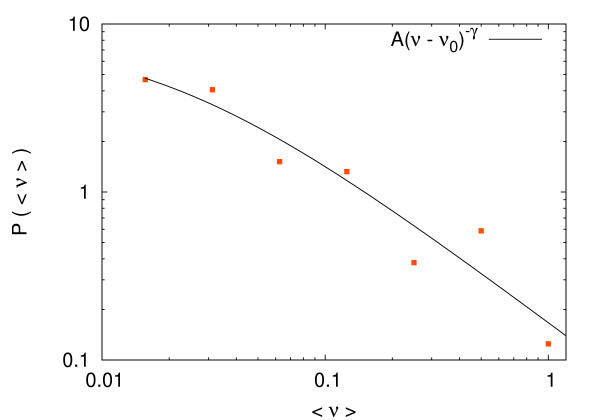
**Distribution of average fluxes**. Distribution of average fluxes (⟨*ν*⟩) of the reactions for the *E. coli *metabolism (points) obtained from the marginal probability distributions computed using our message-passing algorithm. We also display a power-law fit function *P*_fit_*(ν) *= *A*/(*ν *+ *ν *0)*γ *(solid line) with *ν*_0 _= 0.0002 and *γ *= 1.48.

We ran our algorithm on this network and computed the average fluxes in each reaction. We have performed a statistical analysis of these averages in the spirit of [14,24,25]. The probability distribution function (pdf) of these average values is displayed in Figure 11. As can be clearly seen the distribution is large and may be fitted with a power law distribution of *P*(*ν*) ∠ (*ν *+ *ν*_0_)^-*γ*^. The fit gives an exponent *γ *around 1.5, a result that compares very well with previous simulations found in: (i) [14] where FBA optimal fluxes were averaged over many different external condition, (ii) [24] where a maximal local-output strategy is used, and (iii) [25] where a Gaussian approximation for the marginal flux distributions is used. It is claimed in [14] that the robustness of this value *γ *is a signature of universality in cell's metabolism.

**Figure 12:**
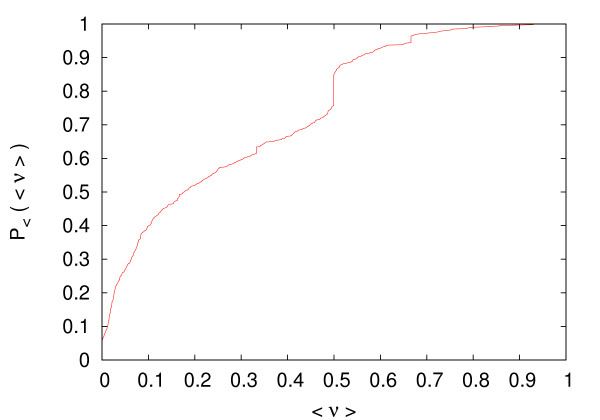
**Integrated distribution of average fluxes**.Integrated distribution *P*_<_of average fluxes ⟨*ν*⟩ of reactions in *E. coli *metabolism obtained from the marginal probability distributions computed using our message-passing algorithm. Note the jumps for ⟨*ν*⟩ = 0.4, ⟨*ν*⟩ = 0.5 and ⟨*ν*⟩ = 0.6.

A more careful analysis of the data may reveals however that the distribution of averages fluxes has a richer structure. In Figure 12 we present the cumulative distribution function $P_{<}(\nu )\equiv \int_{-\infty }^{\nu }P(y)dy$ of theaverage fluxes of the reactions and a clear jump appears for *ν *≈ 0.5, and smaller ones for *ν *≈ 0.4 and *μ *≈ 0.6. At present we do not know whether these jumps are just due to statistical fluctuations (and are correctly smeared out in the usual binning process done to plot the pdf in double logarithmic scale) or they reflect relevant biological or structural information about the network.

## Conclusion

We proposed a novel algorithm to estimate the size and shape of the affine space of a non full-dimensional convex polytope in high dimensions. The algorithm was tested in specific benchmark, i.e. random diluted stoichiometric matrices at a given ratio *α *= *M/N *and fixed number of terms different from zero *K*, in each of the reactions, with results that compare very well with those of exact algorithms. Moreover, we show that while the running time of exact algorithms increases more than exponentially for already moderate sizes, our algorithm is polynomial. The program was run on the Red Blood Cell metabolism producing with shorter computational time results that are in both quantitatively and qualitatively in good agreement with those obtained by MCS presented in [10,11].

Then, our program was used to study the *E. coli *central metabolism, and as expected, reactions with little redundancy turned out to be the ones with larger impact in the size of the space of the metabolic solutions. Specifically, most of the reactions associated with the transformation of glucose in pyruvate, belong to this set, as well as some reactions in the citric cycle. In addition we show strong correlations between the characteristics of the flux distributions of the wild type network and the changes in size of the space of solutions after reaction knock-outs. Finally, we calculate the distribution of the average values of the fluxes in the metabolism of the *E. coli *and present results that are consistent with those of the literature. In the present approach we have mainly followed a discretization strategy that although polynomial, becomes computationally rather heavy for mass balance equation containing a large number of fluxes. We are currently investigating other representation schemes for the messages. The final hope is to obtain an algorithm that allows for on-line analysis of organism-wide metabolic networks.

Let us conclude by noting that in principle the presented approach can be extended to deal with constraints whose functional form is more general than linear, provided that the number of variables involved in each of the constraints remains small, as in the case of inequalities enforcing the second law of thermodynamics for the considered reactions [26]. Work is in progress also in this direction.

## Methods

### Belief Propagation

In the previous section we have seen how the metabolic problem can be cast into a constraint satisfaction framework where each of *M *mass-balance equations imposes a constraint onto a subset of the metabolic fluxes. Let *A *be the set of equations and *I *the set of fluxes. Consider the *a*-*th *row of $\overset{‸}{S}$, and let {*i *∈ *a*} ≡ {*i*_1_, ..., $i_{n_{a}}$} ⊂ *I *be the labels of the fluxes involved in the considered equation having stoichiometric coefficients different from zero. Let also {*a *∈ *i*} ≡ {*a*_1_, ..., $a_{n_{i}}$} ⊂ *A *be the labels of the equations in which flux *i *participates. The emerging structure is a bipartite graph, with two types of nodes: *variable *nodes representing the fluxes of the reactions and *factor *nodes imposing mass conservation. In this case marginals become *q*-modal probability densities that for large values of $q_{i}^{\max}$ will approximate better and better the continuous set of probabilities.

Under the hypothesis that the factor graph is a tree it can be shown [19,27] that a given flux vector ***ν ***satisfying all flux-balance constraints can be expressed as a product of flux and reaction marginals [19-21]:

(8) $$P(\nu )=\prod_{a\in A}P_{a}({\left\{\nu_{l}\right\}}_{l\in a})\prod_{i\in I}P_{i}{\left(\nu_{i}\right)}^{1-d_{i}}$$

where *d*_*i *_is the number of equations in which flux *ν*_*i *_participates (i.e. the degree of site *i*). The marginal probabilities are defined as:

(9) $$\begin{array}{c} P_{i}(\nu_{i})=\sum_{{\left\{\nu_{j}\right\}}_{j\neq i}}P(\nu )\\ p_{a}({\left\{\nu_{l}\right\}}_{l\in a})=\sum_{{\left\{\nu_{j}\right\}}_{j\neq a}}P(\nu ). \end{array}$$

One can then define an entropy in terms of the marginal probability distributions which amounts to the logarithm of the number of solutions of the associated constraint satisfaction problem. From a more geometrical point of view, this entropy is a count of the (logarithm of) the number of elementary *ε*-cells intersecting the polytope Π (see Figure 3).

(10) S≡−∑νP(ν)ln⁡P(ν)=∑a∈A∑{νj}j∈aPa({νj}j∈a)log⁡Pa({νj}j∈a)−∑i∈I∑νi(di−1)Pi(νi)log⁡Pi(νi)

One may wonder how such an approach could be useful in a *real-world *situation where the graph is not a tree. One can hope that typical loop length are large enough to ensure weak statistical dependence of neighboring sites which lay at the heart of the Bethe approximation [28,29]. It is interesting to note that the Bethe approximation is successfully used in many different problems with loopy graph topologies. This is for instance the case for LDPC error correcting codes in Information Theory [30] used in wireless Internet transmission technologies such as WiMAX, or in many inference problems such as graphical models [31], binary perceptron learning [32], and in constraint satisfaction problems such as Satisfiability or Coloring [33,34]. In all these cases although there is no mathematically rigorous proof about the quality of the solution, whenever the algorithm converges, the result generally provides a good estimate of marginal probability distributions.

The algorithm is based on two type of messages exchanged from variable nodes to functional nodes, and vice versa:

• *μ*_*i *→ *a*_(*ν*): the probability that flux *i *takes value *ν *in the absence of reaction *a*.

• *m*_*a *→ *i*_(*ν*): the non-normalized probability that the balance in reaction *a *is fulfilled given that flux *i *takes value *ν*.

The two quantities satisfy the following set of functional equations:

(11) ma→i(νi)=∑{νl}l∈a\iδ(∑l∈asa,lνl;ba)∏l∈a\iul→a(νl)μi→a(νi)=Ci→a∏b∈i\amb→i(νi)

where $\sum_{{\left\{\nu_{l}\right\}}_{l\in a\backslash i}}$ means the sum over all values of fluxes around metabolite *a *but *i*, *b *∈ *i*\*a*, is the set of metabolites in reaction *i *but *a*, *C*_*i *→ *a *_is a constant enforcing the normalization of the probability *μ*_*i *→ *a*_(*ν*), and *δ *(·; ·) is the Kronecker delta function (*δ *(*a*; *b*) is 1 if *a *= *b *and 0 otherwise). The set of Equations 11 can be solved iteratively and upon convergence of the algorithm one can compute the marginal flux distributions as:

(12) Pa({νl}l∈a)=Ca∑{νl}l∈aδ(∑l∈asa,lνl;ba)∏l∈aul→a(νl)Pi(ν)=Ci∏l∈iml→i(ν)

A brute force integration of the discrete set of equation would be much too inefficient for analyzing large networks, due to the multiple dimensional sum over ${\left\{0,...,q_{l}^{\max}\right\}}_{l\in a\backslash i}$ in the previous equation. The first of Equations 11 includes the computation of the convolution of all *μ*_*j *→ *a *_messages except one, an expression of the form $C_{a\to i}={⊗}_{j\in a\backslash i}\mu_{j\to a}$ (where ⊗ denotes convolution) and requires normally *n*_*a *_- 1 convolutions for each outputting message, i.e. *n*_*a *_(*n*_*a *_- 1) convolutions in total for constraint *a*. In the case of the complete *E. coli *network we have mass-balance equations with *n*_*a *_as large as 500, then reducing the computational complexity of the iteration has a cogent practical implication on the performance of the proposed algorithm. There is a way to reduce this quadratic load to just *O *(*n*_*a*_) convolutions. The method we propose is not confined to convolutions and works generally for any associative operation. Note that for operations that are efficiently invertible and commutative, one could just operate all *n*_*a *_terms (*n*_*a *_initial operations) and then for each outputting message operate with the inverse of the undesired element (just one more operation for each message, totalizing just 2*n*_*a *_operations for constraint a plus invertions) i.e. $C_{a\to i}=\left({⊗}_{j\in a}\mu_{j\to a}\right)⊗\mu_{i\to a}^{-1}$. When elements are not invertible, nor conmutative or are just difficult to invert, the following more general scheme can be applied. Let us concentrate for instance on metabolite *a *and let us assume that *n*_*a *_is a power of two, say 2^*n *^(this assumption can be easily relaxed). We will iteratively build a *n *× *n*_*a *_auxiliary matrix $h_{i}^{l}$ setting as initial condition $h_{i}^{0}=\mu_{i\to a}$ and then building the following sequence: $h_{i}^{d}=h_{2i}^{d-1}⊗h_{2i+1}^{d-1}$ for *d *= 1, ..., *n *- 1. This is equivalent to operate consecutive pairs of fluxes over metabolite *a*, then consecutive quadruples and so on. One needs just 2*n*_*a *_operations for computing the full matrix at this stage.

**Figure 13:**
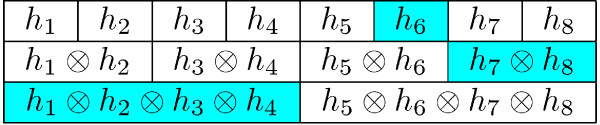
**Linear cavity summation**. An example showing the summation strategy for a metabolite with 8 fluxes. Once all elements of the table are computed (6 convolutions are needed), one needs only 3 more convolutions to compute each one of the 8 cavity messages. In the figure the table elements that are needed to compute ⊗_*j *≠ 5 _*h*_*j *_= (*h*_1 _⊗ *h*_2 _⊗ *h*_3 _⊗ *h*_4_)⊗ (*h*_7 _⊗ *h*_8_) ⊗ *h*_6 _are colored in blue.

To compute *C*_*a *→ *i *_one needs additionally *n *= log_2 _*n*_*a *_operations: we just have to operate the complement of *i *in its pair with the complement of this pair in its quartet and so on on all levels, i.e. in a compact form ${⊗}_{d=0}^{n}h_{[i/2^{d}]\text{xor }1}^{d}$ (See Figure 13). The total number of operations for all out messages becomes then 2*n*_*a *_+ *n*_*a*_log_2 _*n*_*a*_. Moreover, if all messages are to be computed sequentially this number can be further reduced to *O *(*n*_*a*_).

## Authors' contributions

Authors equally contributed to this work. All authors read and approved the final manuscript.

## Supplementary Material

Additional file 1 Human red blood cell metabolism supplementary files. The archive contains 4 text files: rbc.sto (stoichiometric matrix where each column is a reaction and each line is a metabolite), rbc.flu (one column, contains the sign of exchange fluxes: negative are incoming positive outcoming, zero free), rbc.names (contains the name of both reactions and metabolites), and rbc.max (contains maximum flux values of the reactions). Click here for file

Additional file 2 *E. coli *central metabolism supplementary files. The archive contains 3 text files: central.sto (stoichiometric matrix where each column is a reaction and each line is a metabolite), central.flu (one column, contains the sign of exchange fluxes: negative are incoming, positive outcoming, zero free), central.names (contains the name of both reactions and metabolites). Click here for file
